# Transient central hypoxemia due to intermittent high-degree atrioventricular block in a heart-transplanted patient diagnosed during routine electroencephalography: a case report

**DOI:** 10.1186/s13256-022-03574-6

**Published:** 2023-01-06

**Authors:** Matthieu Raboud, Andrea M. Humm, Hari Vivekanantham, Philipp Suter

**Affiliations:** 1 Department of Internal Medicine, University and Hospital of Fribourg, Chemin des Pensionnats 2-6, 1708 Fribourg, Switzerland; 2 Neurological Unit, Department of Internal Medicine, University and Hospital of Fribourg, Fribourg, Switzerland; 3 Department of Cardiology, University and Hospital of Fribourg, Fribourg, Switzerland; 4 Department of Pulmonary Medicine, University Hospital and University of Bern, Bern, Switzerland

**Keywords:** High-degree atrioventricular block, Heart transplantation, Cerebral hypoxemia, Cerebral hypoperfusion

## Abstract

**Background:**

Bradycardia frequently occurs in heart-transplanted patients, mainly as a temporally restricted manifestation early after transplantation and often without symptoms. A high-degree atrioventricular block is mostly symptomatic through cerebral hypoxia induced through cerebral hypoperfusion. Only a few published cases show this specific electroencephalography result in this context. The purpose of this case is to bring attention to atypical manifestations of typical cardiac complications after heart transplantation and the importance of perseverance in the diagnostic.

**Case presentation:**

A Central European man in his 50s with history of heart transplantation 31 years previously was admitted to the internal medicine ward for short-lived recurrent episodes of generalized weakness with multiple falls but without loss of consciousness. During routine electroencephalography, the patient perceived this recurrent sensation. This episode coincided with a transient third-degree atrioventricular block followed 8–10 seconds later by a generalized slowing of the electroencephalography, reflecting cerebral hypoxia due to cerebral hypoperfusion. Holter monitoring confirmed the diagnosis. A pacemaker was implanted, consequently resolving the episodes.

**Conclusion:**

This case report illustrates the pathophysiological central hypoxemic origin of episodes of generalized weakness caused by a high-degree atrioventricular block in a patient surviving 29 years after heart transplant. It highlights the benefit of electroencephalography as a diagnostic tool in well-selected patients.

## Background

Bradycardia after heart transplantation has been well documented for decades. It occurs in at least 40% of recipients in the early days following heart transplantation, and it is usually a temporally restricted occurrence [1]. Expert opinions recommend a wait-and-see or medical approach, but temporary pacemakers are sometimes required [2–4]. Preoperative use of amiodarone is probably the leading cause of this complication [5].

Bradycardia is rarely seen during long-term follow-up after heart transplantation. It is primarily due to sinus node dysfunction and rarely high-degree AV blocks [2, 3]. The causes of these complications are not well understood. However, they include surgical trauma to the conduction system (sinoatrial and atrioventricular nodes) or transplant arteriopathy [6].

Bradycardia has been mainly described as an intermittent condition that can either be symptomatic or not. Symptoms are due to cerebral hypoxia induced by cerebral hypoperfusion [7]. Frequent symptoms range from fatigue, unspecific generalized weakness, mild orthostatic dizziness, to syncope, the latter eventually associated with hypoxic myoclonic jerks [4, 7, 8].

Most cases only present an intermittent or paroxysmal block, making the diagnostic process difficult [8].

Pacemaker implantation is the treatment of choice for symptomatic high-degree nonreversible AV blocks following heart transplantation [2, 3].

## Case presentation

A Swiss man in his 50s was hospitalized after a fall at home caused by short-lived recurrent episodes of generalized weakness, causing several pelvic ring fractures. He had an orthotopic heart transplantation in 1991 due to idiopathic dilatative cardiomyopathy and non-anticoagulated paroxysmal atrial fibrillation. The longstanding immunosuppressive treatment caused both chronic kidney disease stage G5A3 after KDIGO and middle and lower lobe pulmonary carcinoma in 2006, treated by right pneumonectomy. He has required kidney dialysis for over 20 years, is under cyclosporin and mycophenolate mofetil, and has no known allergies. The patient is a former salesman.

Regarding the fall leading to the fractures and hospitalization, the patient described a loss of control of his limbs for a few seconds, without a perceived loss of consciousness. These episodes had occurred over the previous 8 years (since 2012) but had become more frequent, at that time reaching three or four times a week. The patient could not identify any triggering factors, and the episodes could occur at rest or during effort. He did not have memory gaps, altered consciousness after weakness episodes, a bitten tongue, or urinary incontinence. He did not report any thoracic pain, dyspnea, or palpitations.

### Investigations

Blood analysis revealed an elevated creatinine level (creatinine 513 μmol/l, urea 8.6 mmol/l) and anemia (hemoglobin 86 g/l) due to terminal renal failure. However, no other abnormalities such as electrolytic disturbances, thyroid dysfunction, or inflammatory syndrome were observed. Protein electrophoresis and immunofixation were normal for a patient with renal failure. Radiological examinations revealed three nondisplaced pelvic ring fractures: right ischiopubic ramus, right iliopubic ramus, and left sacral ala. First, we suspected the cause to be a cardiac one because of the long-term transplant. The patient had regular cardiac follow-ups with multiple coronary angiograms, Holter monitoring, and cardiac ultrasounds without any significant abnormalities except for high-burden atrial fibrillation and a left ventricular ejection fraction of 50%. In line with the patient’s choice, paroxysmal atrial fibrillation (CHA2DS2-VASc 2 points, HAS-BLED 1 point) had not been anticoagulated in the context of frequent epistaxis due to Rendu–Osler disease.

**Fig. 1:**
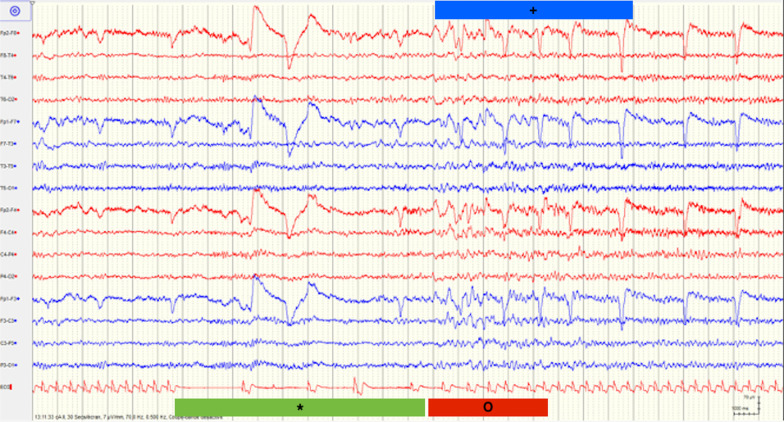
Electroencephalogram with third-degree AV block during 10 seconds (green*) and corresponding mild cerebral hypoxia (red+) and patient typical sensation (blue+) during 5 seconds

**Fig. 2:**
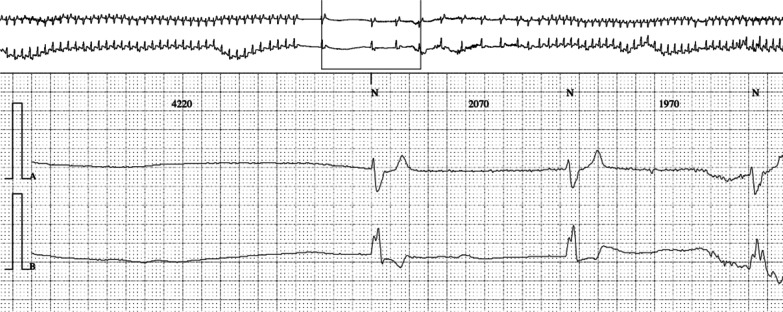
Holter electrocardiogram with sinus arrest and ventricular escape rhythm

**Fig. 3:**
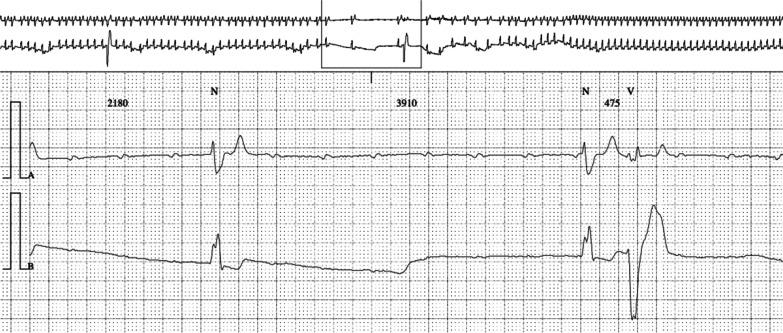
Holter electrocardiogram with third-degree atrioventricular block and ventricular escape rhythm

**Fig. 4:**
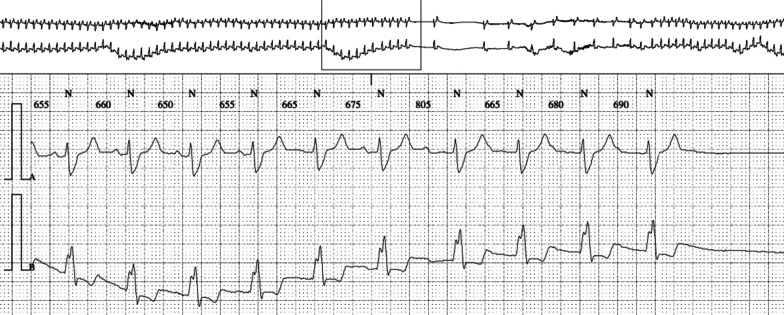
Holter electrocardiogram with second-degree atrioventricular block of Mobitz type 1 (Wenckebach)

The entry electrocardiogram was normal, and 72-hour Holter monitoring was pending. We also considered an orthostatic cause, but a Schellong test could not be performed due to fractures. A typical episode was recorded during a routinely performed EEG of 20 minutes (Fig. 1), where we identified a 10-second period with transient third-degree AV block on the electrocardiogram trace. Eight to ten seconds after the beginning of cardiac arrhythmia, the EEG (10–20 system) showed mild generalized theta–delta slowing, compatible with mild hypoxic encephalopathy. More or less at the same time, cardiac arrhythmia stopped spontaneously, and EEG normalized within 5 seconds. The patient confirmed having a typical subjective episode, which coincided with the few seconds of EEG slowing. In the meantime, the Holter monitoring confirmed the diagnosis of transient third-degree AV block with bradycardia and a ventricular escape rhythm and several episodes of second-degree AV block of Mobitz type 1 (Wenckebach) (Figs. 2, 3, and 4). The longest sinus arrest was 4220 ms with a ventricular escape rhythm.

### Differential diagnosis

The differential diagnosis in our patient was quite broad. Initially, we did not consider cardiac investigations, as the patient already had regular cardiac follow-up, reported to be within normal limits except for paroxysmal atrial fibrillation. We completed the investigations with a neurologic evaluation, including EEG, even though neurological differential diagnoses such as neuromuscular disease or epileptic seizures were not at the top of the list.

### Treatment

We retained the indication for a single-chamber ventricular pacemaker (Medtronic Astra XT SR MRI X2SR01, right ventricular lead Medtronic 5076-58 CapSureFix Novus 58 cm) with VVI stimulation. We rejected a dual-chamber pacemaker due to a high burden of paroxysmal atrial fibrillation, and the patient refused Micra implantation. The pacemaker was placed in the right prepectoral position with the right subclavian approach. The procedure was performed without any complications.

The pelvic ring fractures were treated conservatively.

### Outcome and follow-up

No recurrent episodes occurred during follow-up. The patient remained asymptomatic, and device interrogations showed adequate anti-bradycardia pacing. A specific etiology for the third-degree AV block was not identified.

An ischemic process was suspected but reasonably excluded by coronary angiogram, which showed no significant atherosclerosis or vascular lesion requiring revascularization. No recent cardiac procedure could explain a potential iatrogenic cause of AV block. The patient did not take any medication with a negative dromotropic effect nor any drugs associated with occurrences of complete heart blocks. The presentation was not consistent with myocarditis. We did not find any clinical argument for graft rejection.

A transient third-degree AV block had been discovered accidentally under cardiac monitoring in a previous hospitalization for atrial fibrillation 5 years previously. This episode had been attributed to the ongoing and recently introduced amiodarone treatment. Therefore, the medication had been stopped and not readopted. Follow-up Holter monitoring revealed no cardiac arrhythmias except paroxysmal atrial fibrillation.

## Discussion and conclusion

We present herein the case of a heart-transplanted patient with short-lived recurrent episodes of generalized weakness. The initial cardiac follow-up consisted of coronary angiograms, Holter monitoring, and cardiac ultrasounds; only high-burden atrial fibrillation was observed. Finally, the realized EEG identified central hypoxia with a high-degree AV block as the etiology. We decided to implant a single-chamber ventricular pacemaker, and no further episodes were observed in follow-up.

As mentioned above, late-onset AV blocks in patients with orthotopic heart transplantation are rare, and their pathophysiology is not well understood. The diagnosis is often difficult because of its primarily intermittent presentation [8]. Studies have shown that the incidence of AV blocks requiring implantation of a pacemaker at any time is 7.5%, with 3.3% occurring in the first 3 months after transplantation, while only 2.1% are late-onset high-degree AV blocks [2, 8].

Cardiac rejection has also been well demonstrated as a cause of AV blocks, especially in the acute phase (< 3 months) post transplant due to the involvement of the conduction system. To our knowledge, infiltration of the conducting system has never been documented in chronic rejection (> 3 months) [9–11].

The most common cause of later death after heart transplantation is graft coronary artery disease with chronic coronary insufficiency with repercussions on the conduction system, which was not the case in our patient with healthy coronaries [9, 10].

The cause of late-onset AV blocks is not yet known. Studies do not point to any correlation with the operation time, the donor’s age, or the age of the transplanted heart [8]. Due to the age of the transplanted heart, a fibrotic process is most probably the primary origin.

Diagnostic work-up was not straightforward in our case. Initially, we suspected low blood flow caused by decreased cardiac output due to the paroxysmal atrial fibrillation bradycardia. We, therefore, decided on cardiac Holter monitoring. Indeed, an arrhythmia caused the recurrent short-lived episode of generalized weakness.

In literature, only two similar high-degree AV blocks have been diagnosed during EEG, a third-degree and a second-degree type Mobitz II. In those cases, the profound cerebral hypoxia provoked a convulsive seizure [12].

Typical central nervous system complications after solid-organ transplantation include immunosuppressor-induced neurotoxicity, central nervous system infections, cerebrovascular disease, and different types of epileptic seizures, which are usually tonic–clonic with focal onset or primarily generalized [13, 14]. The incidence of epileptic seizures depends on the transplanted organ and reaches between 2% and 20% after orthotopic heart transplantation [14]. Mostly, they appear in the early period after transplantation and are due to cerebral ischemia secondary to perioperative hemodynamic instability or due to metabolic disorders [14, 15]. Patients with end-stage heart failure before transplantation may have chronically decreased brain perfusion, making them particularly vulnerable to encephalopathy and, consequently, seizures [14]. However, most epileptic seizures are provoked by the immunosuppressive treatment, especially calcineurin inhibitor treatment such as cyclosporin or tacrolimus. Above all, abrupt increase in the dosing of immunosuppressive medication or abnormally high blood levels favor seizure provocation. Other immunosuppressive treatments including antiproliferative treatments such as mycophenolate mofetil or azathioprine or corticosteroids are much less likely to provoke epileptic seizures [13]. Calcineurin-inhibitor-triggered epileptic seizures may be explained by the inhibition of the gamma-aminobutyric acid system leading to increased and potentially synchronized neuronal transmission and hence to an epileptic seizure [14].

The diagnosis of seizures in transplant patients is no different from that in other patients suffering from epileptic fits, and this applies also largely to the treatment goals, given the absolute necessity for long-term immunosuppression. However, special attention must be paid to drug–drug interactions, enzyme induction, or tolerability. Newer antiepileptic drugs have a more favorable adverse effect profile and fewer drug interactions than older drugs. A specialist should always be consulted when prescribing or adapting antiepileptic drugs in patients with solid-organ transplantation [13].

We considered a primary epileptic cause in the initial differential diagnosis of our patient. However, the clinical presentation was not typical for focal with secondary generalization or focal to bilateral tonic–clonic seizures (no post-ictal phase, no loss of urine or stool, and no bitten tongue). Primary generalized or nonmotor seizures (“absence seizures”) could be considered but would be rather unlikely given the first manifestation at age > 50 years.

Nevertheless, EEG is a simple, noninvasive investigation that can help in the diagnostic workup, especially in atypical clinical situations. However, EEG should not be systematically performed in all patients with transient loss of consciousness and syncope because only 1–2 % of EEGs show potentially epileptic potentials. Meanwhile, the cost for one diagnostic-relevant EEG is up to US $33,000 [16, 17]. Our patient suffered from recurrent episodes of presyncope, subjectively perceived as short-lived generalized weakness and sometimes associated with falls. There was no complete syncope with evident loss of consciousness, as seen in more sustained bradyarrhythmia due to a high-degree AV block. It is possible that our patient did not suffer from typical vasovagal episodes because of a transplanted and hence vagally denervated heart.

Most syncopes are described in the context of tachyarrhythmias such as ventricular tachycardia or fibrillation [18]. The patient’s symptoms typically appear within 10 seconds after the arrhythmia and are sometimes followed by transient electrocerebral silence [7]. Most published studies about bradycardia-associated cerebral hypoxia due to cerebral hypoperfusion demonstrate that they are induced by ocular compression, carotid sinus massage, or intentional transitional deactivation of an automatic implantable cardioverter–defibrillator [7].

## Take-home messages

- Late-onset atrioventricular block is uncommon following heart transplantation. Its diagnosis can be challenging due to its primarily intermittent nature. Its cause is also not well elucidated yet.
- EEG is a noninvasive, easily available, and cheap diagnostic tool, especially in atypical clinical situations, but the patients who will benefit from this diagnostic workup must be well selected, because of its very low predictive value.
- It is essential to give a good description of the problem to the person in charge of the EEG reading so that unexpected and/or more subtle alterations are not overlooked.
- Epileptic seizures are typical nervous system complications of immunosuppressors, especially calcineurin inhibitors such as cyclosporin or tacrolimus.

## Data Availability

The data that support the findings of this study are available on request from the corresponding author.

## Abbreviations

AV: Atrioventricular
EEG: Electroencephalography
